# Supplementary material for: Layer-by-layer biofunctionalization of nanostructured porous silicon for high-sensitivity and high-selectivity label-free affinity biosensing
Source: Nat Commun. 2018 Dec 10;9:5256. doi: 10.1038/s41467-018-07723-8 (PMC6288083; doi:10.1038/s41467-018-07723-8)
Supplement: Supplementary file 1 — Supplementary Information [file 41467_2018_7723_MOESM1_ESM.pdf]

Supplementary Information

Layer-by-layer biofunctionalization of nanostructured porous silicon for high-sensitivity and high-selectivity label-free affinity biosensing

Stefano Mariani[1], Valentina Robbiano[1], Lucanos M. Strambini[2], Aline Debrassi[3], Gabriela Egri[3], Lars Dähne[3] and Giuseppe Barillaro[1,2]*

[1]*Dipartimento di Ingegneria dell'Informazione, Università di Pisa, Via G. Caruso 16, 56122 Pisa, Italy*

[2]*Istituto di Elettronica e di Ingegneria dell'Informazione e delle Telecomunicazioni, Consiglio Nazionale delle Ricerche, via G. Caruso 16, 56122, Pisa, Italy*

[3]*Surflay Nanotec GmbH, Max-Planck-Straße 3, 12489 Berlin, Germany*

**giuseppe.barillaro@unipi.it*

Content

This supplementary information reports additional figures and data on: morphological and optical characterization of PSi interferometers before and after LbL biofunctionalization; bright field and fluorescence images of control PSi interferometers; stability and specificity of PSi interferometers LbL-biofunctionalized for biotin/streptavidin affinity biosensing; evaluation of PMAA biotinylation degree on the biosensing properties of PSi interferometers; silane-based covalent biofunctionalization of PSi interferometers; LbL assembly stability and unspecific interaction in human saliva; main steps of the Interferogram Average over Wavelength Reflectance Spectroscopy.

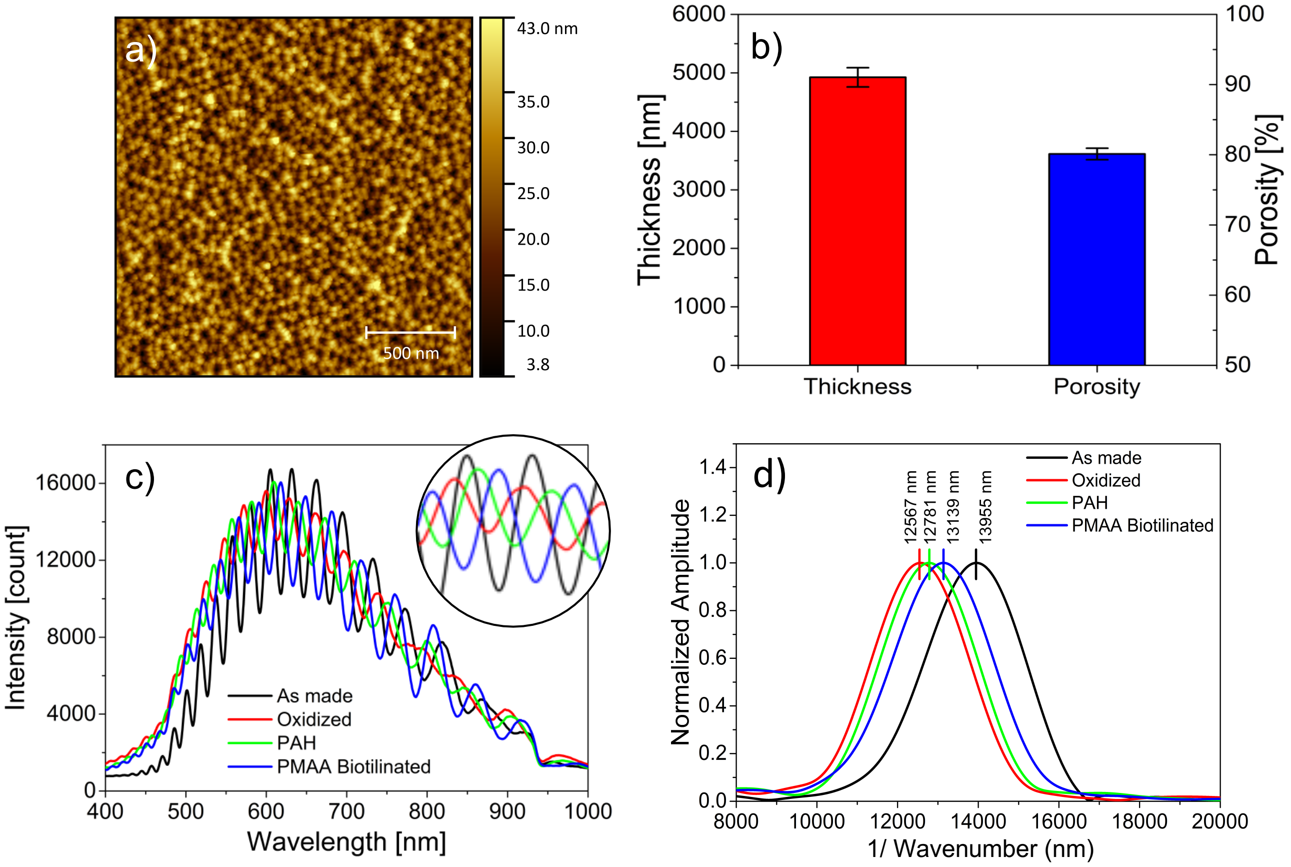

Supplementary Figure 1. Morphological and optical characterization of as-prepared and LbL-biofunctionalized PSi interferometers. (a) AFM top-view image of as-prepared PSi interferometer (scale bar is 500 nm). (b) Thickness (4.92 ± 0.16 µm, red) and porosity (80.1 ± 0.8%, blue) values of as-prepared PSi interferometers. Data are provided as average values over 7 replicates with error bars representing one standard deviation. (c, d) Reflection spectra (c) and FFT amplitude spectra (d) of PSi interferometers as-prepared (black trace), after oxidation (red trace), and after coating with PAH (green trace) and with PAH/b-PMAA (1:30) (blue trace).

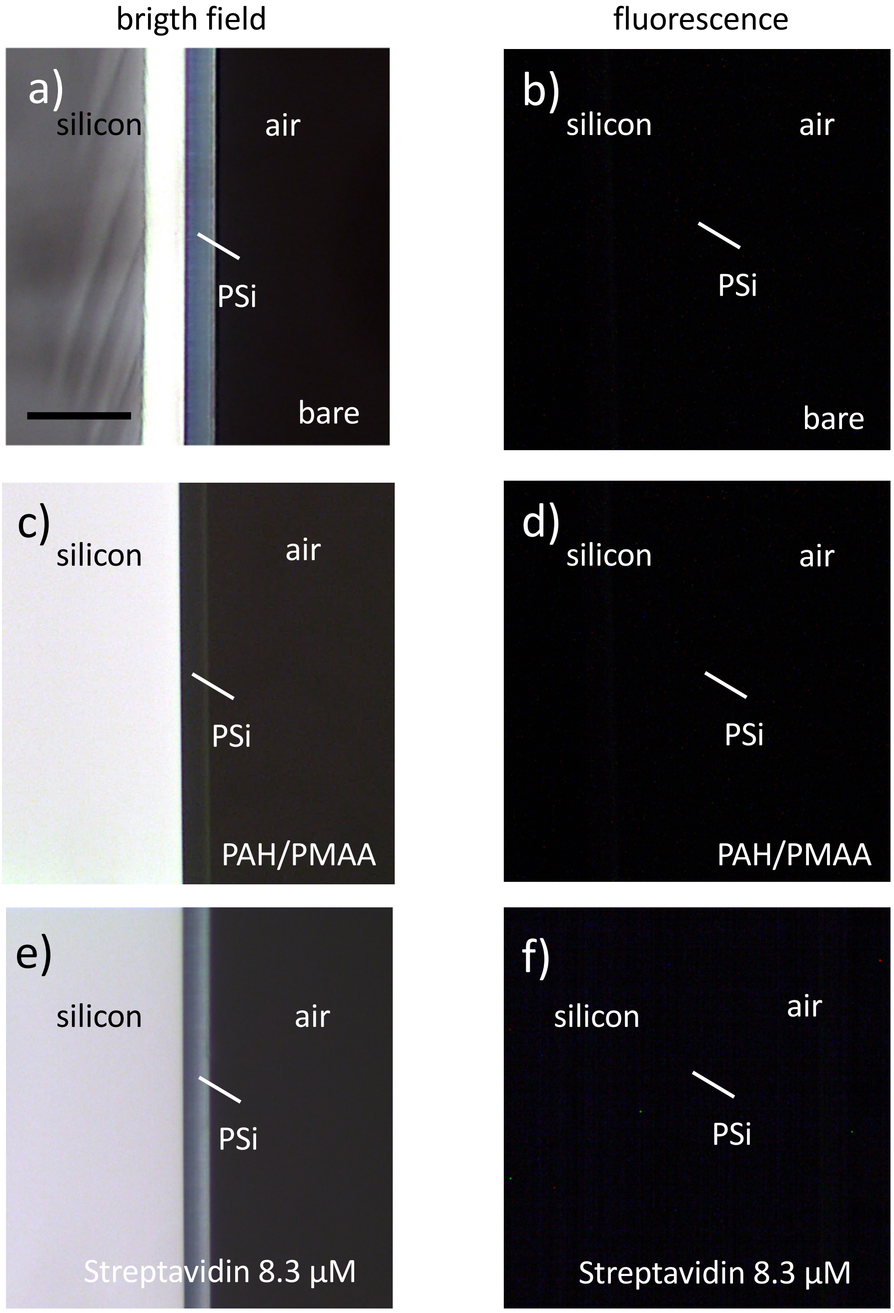

Supplementary Figure 2. Bright field and fluorescence images of control PSi interferometers. (a,b) Cross-section of oxidized PSi interferometers with no LbL coating. (c,d) Cross-section of oxidized PSi interferometers LbL-coated with PAH/b-PMAA (with no fluorophores). (e,f) Cross-section of oxidized PSi interferometers LbL-coated with PAH/b-PMAA, after infiltration with bare (no fluorophores) 8.3 µM streptavidin. Images were collected with a Leica DM2500 M optical microscope. Scale bare is 15 µM.

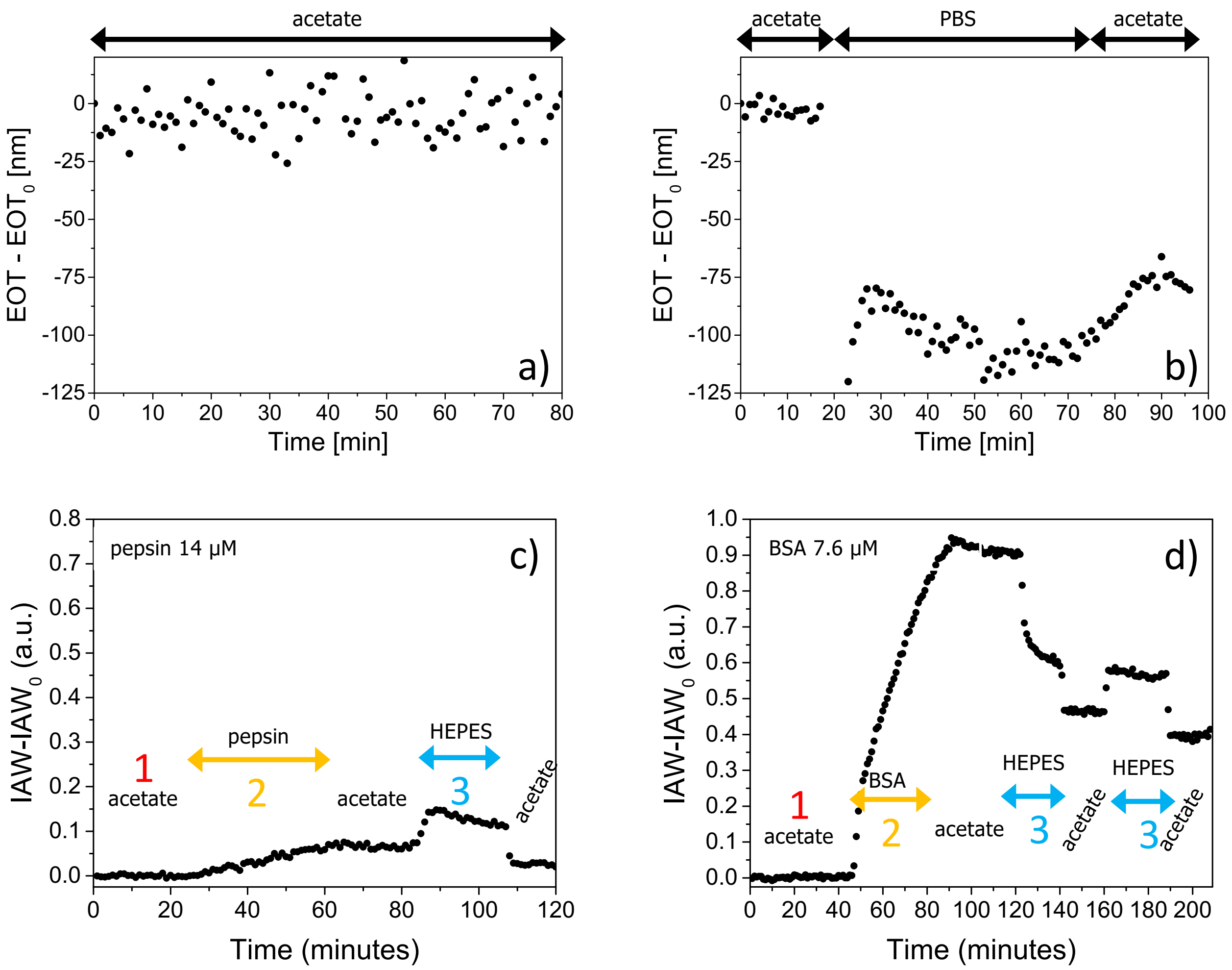

Supplementary Figure 3. Stability and specificity of PSi interferometers LbL-biofunctionalized for biotin/streptavidin affinity biosensing. (a) Sensorgram ($EOT-EOT_0$ vs. time) of LbL-biofunctionalized PSi interferometers highlighting high stability of the LbL nano-assembling in acetate buffer. (b) Sensorgram ($EOT-EOT_0$ vs. time) of LbL-biofunctionalized PSi interferometers highlighting poor stability of the LbL assembly in PBS buffer. (c) Sensorgram ($IAW-IAW_0$ vs. time) of LbL-biofunctionalized PSi interferometers upon injection of pepsin (i.e. 500 µg ml^{-1}, ~ 14 µM) as non-target protein. (d) Sensorgram ($IAW-IAW_0$ vs. time) of LbL-biofunctionalized PSi interferometers upon injection of BSA (i.e. 500 µg ml^{-1}, ~ 7.6 µM) as a worst-case non-target protein.

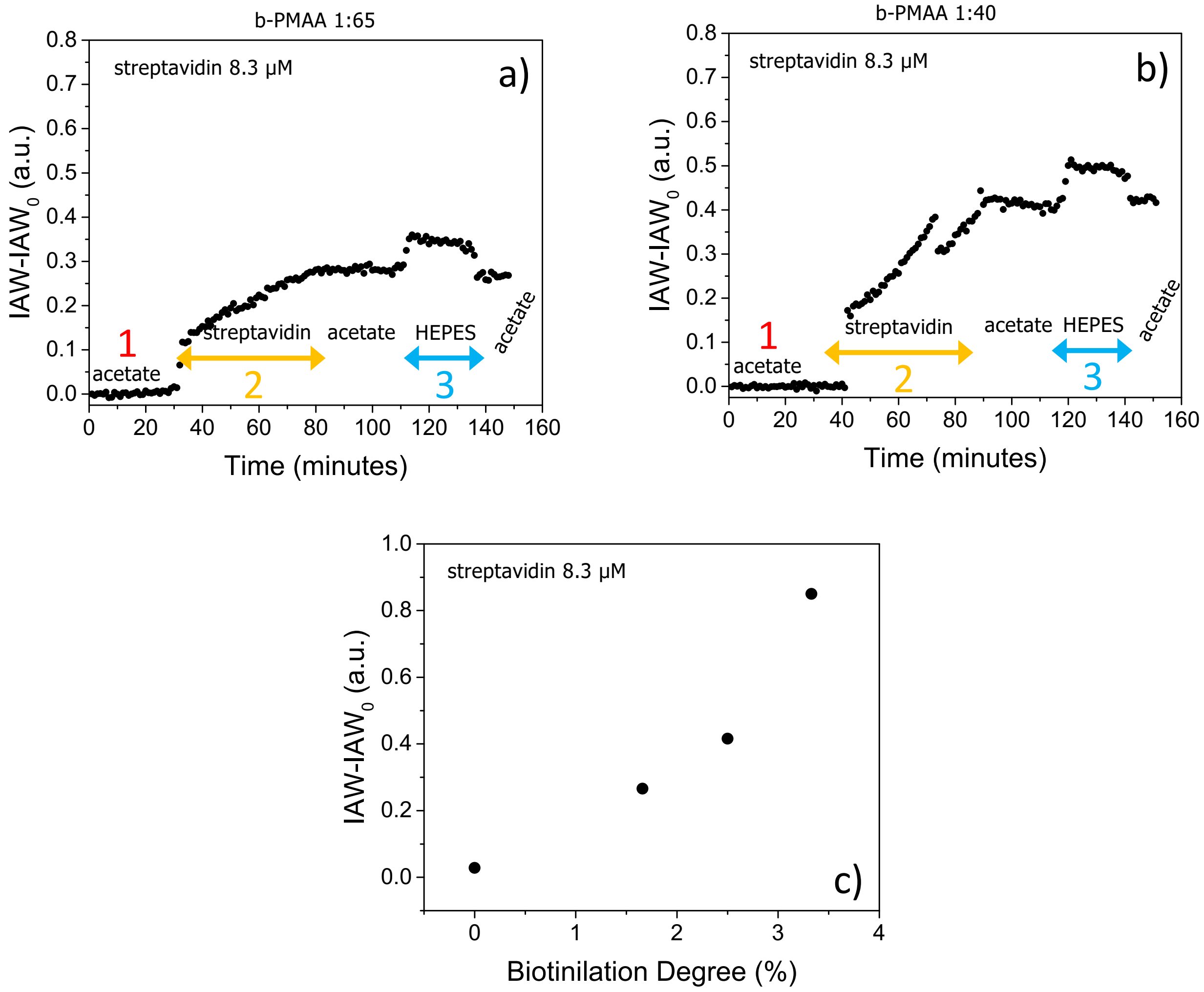

Supplementary Figure 4. Evaluation of PMAA biotinylation degree on the biosensing properties of PSi interferometers. (a,b) Sensorgrams ($IAW-IAW_0$ vs. time) recorded upon injection of 8.3 µM streptavidin (i.e. 500 µg ml^{-1}) for LbL-biofunctionalized PSi interferometers with (a) b-PMAA (1:65) and (b) b-PMAA (1:40). (c) $IAW-IAW_0$ values recorded in acetate buffer at the end of the biosensing protocol for PSi interferometers LbL-biofunctionalized with PMAA at different biotinilation degrees, namely 0% (i.e. bare PMAA), 1.5% (i.e. b-PMAA 1:65), 2.5% (i.e. b-PMAA 1:40), and 3.3% (i.e. b-PMAA 1:30).

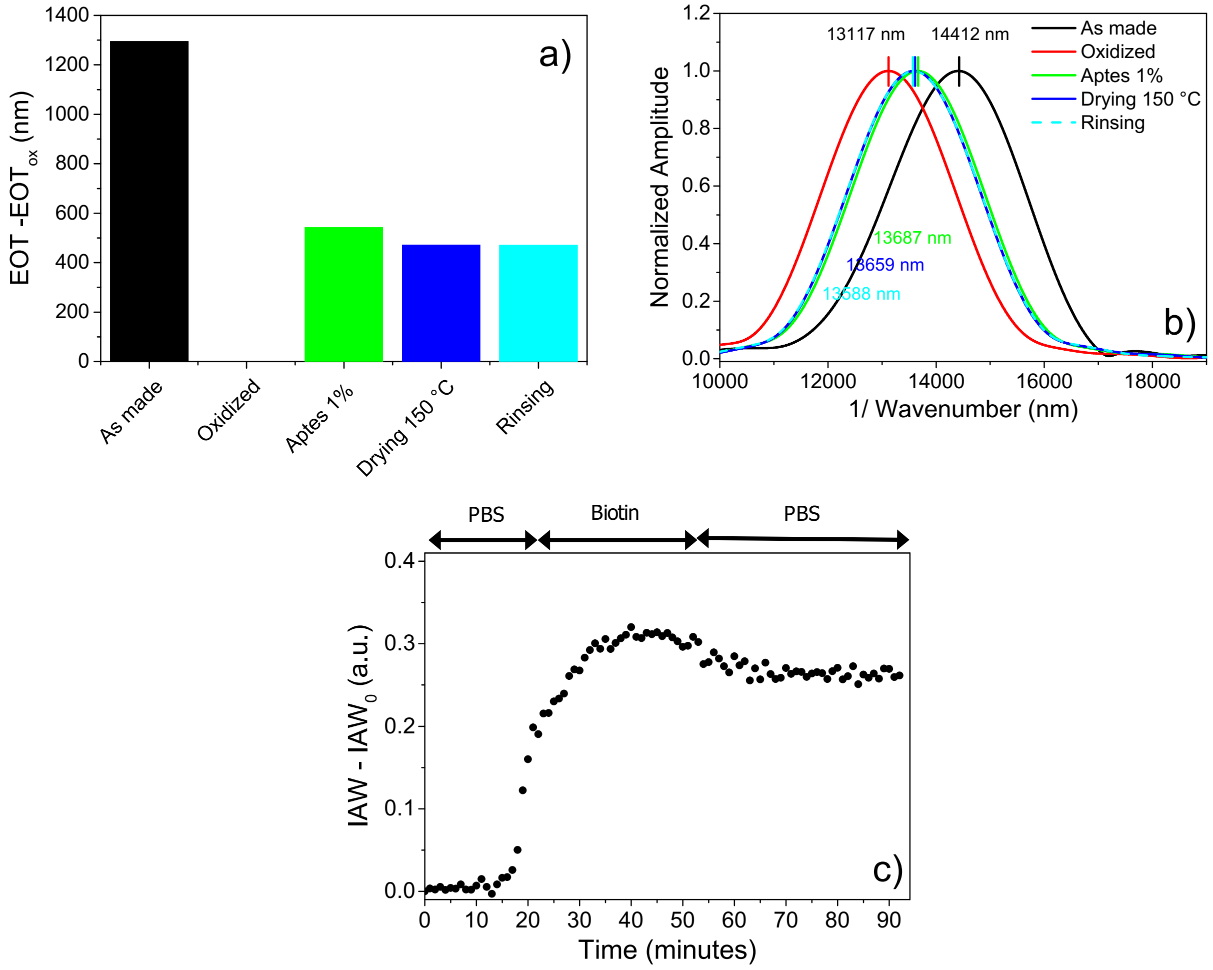

Supplementary Figure 5. Silane-based covalent biofunctionalization of PSi interferometers. (a,b) EOT-EOT_{ox} values (a) and FFT amplitude spectra (b) recorded in air for PSi interferometers as-prepared, as well as after each functionalization step, namely after immersion in 1% APTES in toluene, after baking at 150 °C, and after rinsing. The EOT value of oxidized PSi interferometers (i.e. EOT_{ox}) is used as a reference in (a) to obtain positive differential EOT values for each functionalization step. The peak position along the x-axis in b) represents the effective optical thickness (EOT) value. (c) Sensorgram (IAW-IAW_0 vs. time) recorded for the silanized PSi interferometer in (a,b) during the covalent linking of 3-sulfo-N-hydroxysuccinimide ester sodium salt to aminogroups of the APTES.

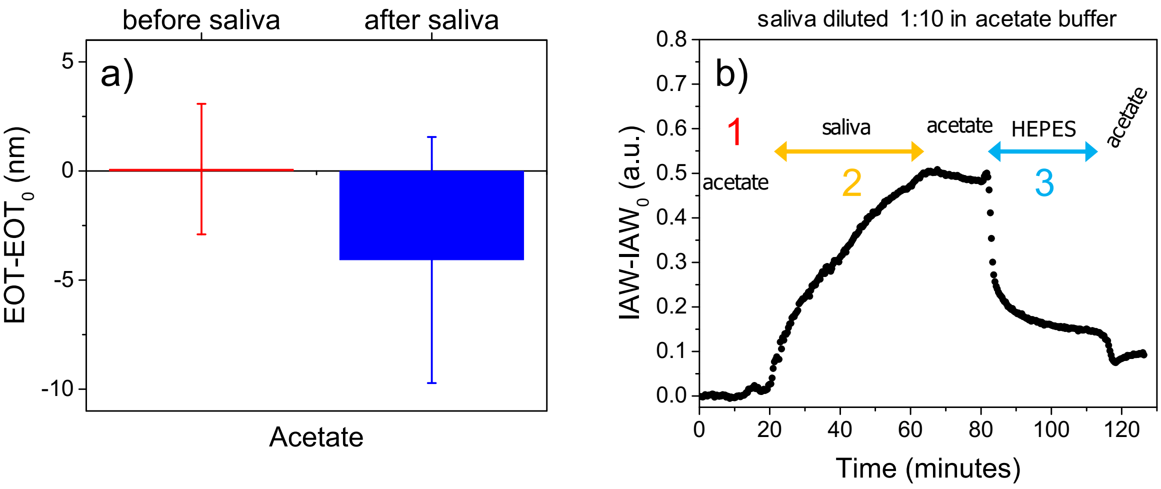

Supplementary Figure 6. LbL assembly stability and unspecific interaction in human saliva. a) $EOT\text{-}EOT_0$ values recorded in acetate buffer before (i.e., 0.08 ± 2.98 nm) and after (i.e., -4.1 ± 5.6 nm) saliva injection (diluted in acetate buffer). The results confirm a good stability of the LbL nanoassembly. Indeed, the ΔEOT values are not statistically different (Student's *t*-test confidence level = 99%) from those recorded injecting acetate buffer over 80 min (i.e., -3.8 ± 12.6 nm, Figure 3b in the manuscript). Data are provided as average values over 3 replicates with error bars representing one standard deviation. b) Sensorgram (i.e. $IAW\text{-}IAW_0$ vs. time) recorded upon injection of diluted saliva, from which it is apparent that biomolecules (present in saliva) unspecifically adsorbed on the LbL coating after saliva injection are efficiently removed through the repulsive rinsing step in HEPES buffer. The residual IAW value (in acetate buffer, over three replicates) after saliva injection is $IAW\text{-}IAW_0 = 0.090 \pm 0.013$ a.u..

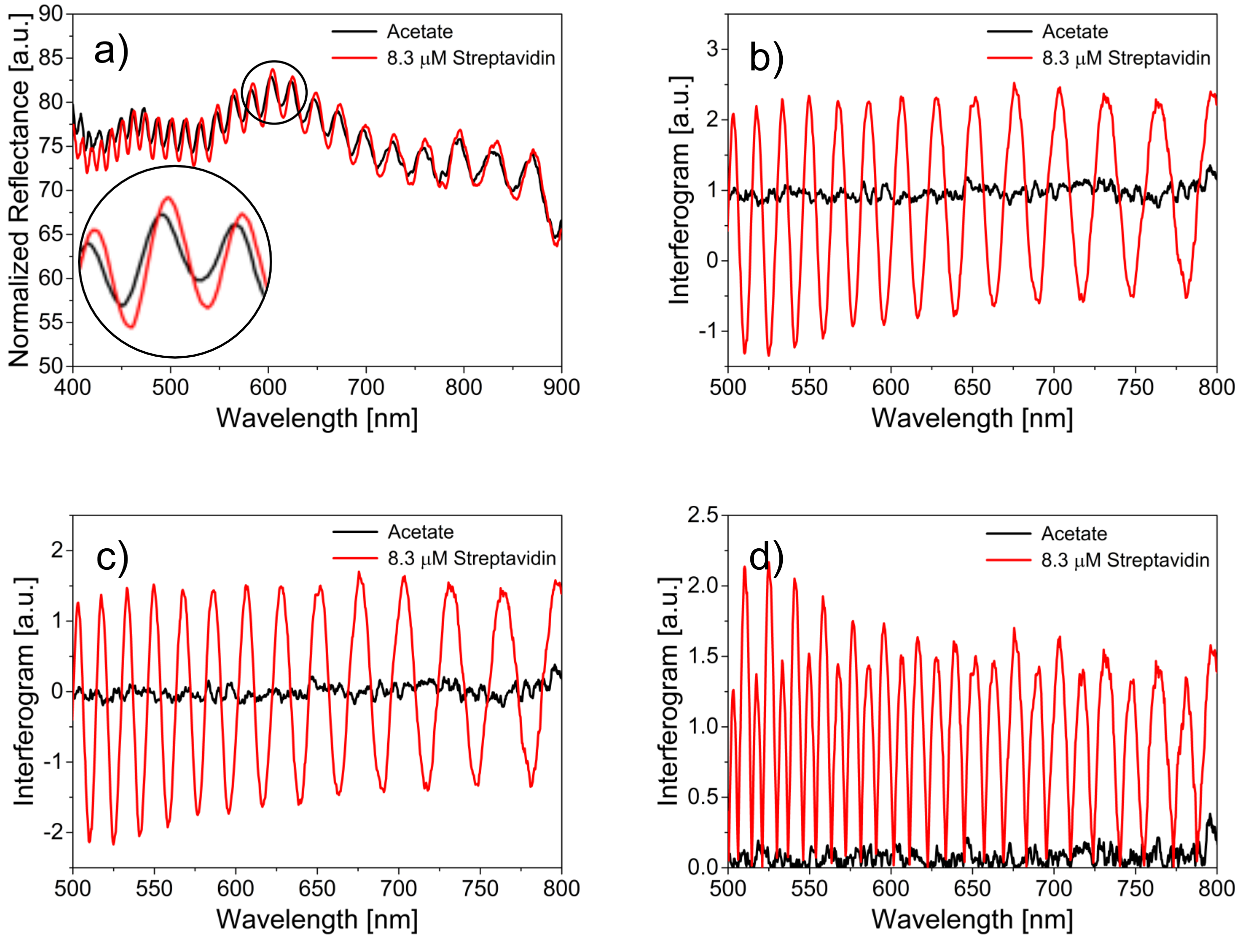

Supplementary Figure 7. Main Steps of the Interferogram Average over Wavelength Reflectance Spectroscopy. (a) Normalized reflectance spectra of PSi interferometers in acetate buffer before (black trace) and after (red trace) injection/binding of 8.3 μM streptavidin. (b−d) Spectral interferograms recorded in acetate buffer before (black trace) and after (red trace) injection of 8.3 μM streptavidin, calculated over the spectral range 500−800 nm, (b) after subtraction of the reflection spectra in (a) from the reference reflection spectrum recorded in acetate buffer at the end of the warm up time; (c) after removal of the average value from the interferograms in (b); (d) after application of the absolute value function to the interferograms in (c).
